# Risperidone Induced DRESS Syndrome: A case report

**DOI:** 10.1192/j.eurpsy.2024.1455

**Published:** 2024-08-27

**Authors:** S. E. Ilgin, Ö. Yanartaş, E. Akça

**Affiliations:** ^1^Psychiatry, Marmara University Research & Training Hospital; ^2^Psychiatry, Marmara University Research & Training Hospital, Istanbul, Türkiye

## Abstract

**Introduction:**

DRESS (Drug Reaction with Eoshinophilia and Systemic Symptoms) syndrome, also called DIHS (Drug-Induced Hypersensitivity Syndrome) is a rare drug-induced systemic hypersensitivity reaction that can be potentially life-threatening (Choudhary et al. J Clin Aesthet Dermatol 2013; 6 -7). Risperidone is an antipsychotic drug with significant antagonist activity at the 5-HT2 and the D2 receptors. It has been reported that risperidone may be effective in controlling agitation, delusion, hallucination, and withdrawal behavior in geriatric patients (Yunusa & El Helou. Front Pharmacol 2020;11:596).

**Objectives:**

The aim of this study is to demonstrate the case of developed DRESS syndrome following the use of risperidone.

**Methods:**

The 81-year-old female patient was admitted to the Dermatology Clinic due to skin rash, high fever and leukocytosis following the use of risperidone. The patient was consulted to Psychiatry.

**Results:**

In her history it was determined that risperidone 0.5 mg/d was started to the patient with depression due to agitative symptom. On the 4th day of treatment, targeoid lesions, starting from the back and spreading first to the trunk and then to the extremities, were observed. Further laboratory examinations revealed that the fever was measured at 39.5 C°, liver enzymes were elevated (ALT= 119 IU/ lt, AST= 124 IU /lt), and significant leukocytosis (WBC =12.000) was present along with the lesions. The patient was planned to be hospitalized to Dermatology Clinic on the 5th day and risperidone was stopped. The patient’s agitation increased and following the risperidone discontinuation thereupon the lesions tended to fade and desquamation began. After the treatment of the DRESS syndrome, aripiprazole was given to the patient for agitative symptom. The level of agitation symptoms decreased, and the patient tolerated aripiprazole well without any observed side effects.

**Image:**

**Figure:**
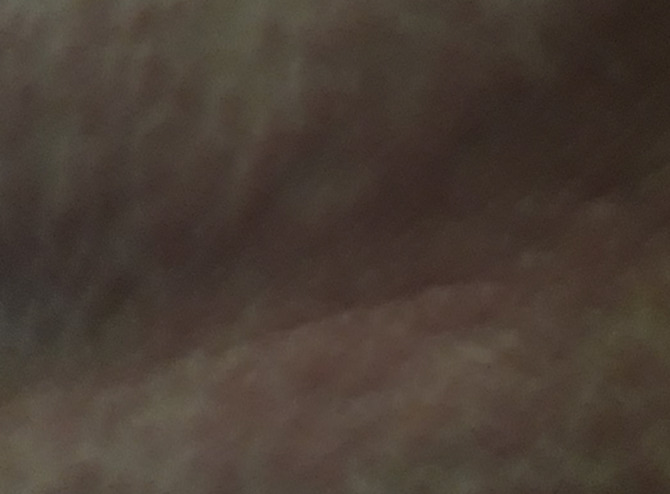

**Conclusions:**

When initiating medication for the elderly population to address agitation, considering such rare side effects can prevent the patient from being hospitalized due to DRESS syndrome. To the best of our knowledge, this is the first case report associated with DRESS syndrome and risperidone treatment thus, it is necessary to be very careful when starting psychotropic medication for elderly patients.

**Disclosure of Interest:**

None Declared

